# Supplementary material for: Changes within the central stalk of E. coli F1Fo ATP synthase observed after addition of ATP
Source: Commun Biol. 2023 Jan 11;6:26. doi: 10.1038/s42003-023-04414-z (PMC9834311; doi:10.1038/s42003-023-04414-z)
Supplement: Supplementary file 2 — Supplementary Information [file 42003_2023_4414_MOESM2_ESM.pdf]

SUPPLEMENTARY INFORMATION

Changes within the central stalk of *E. coli* F_1F_o ATP synthase observed after addition of ATP

Meghna Sobti et al.

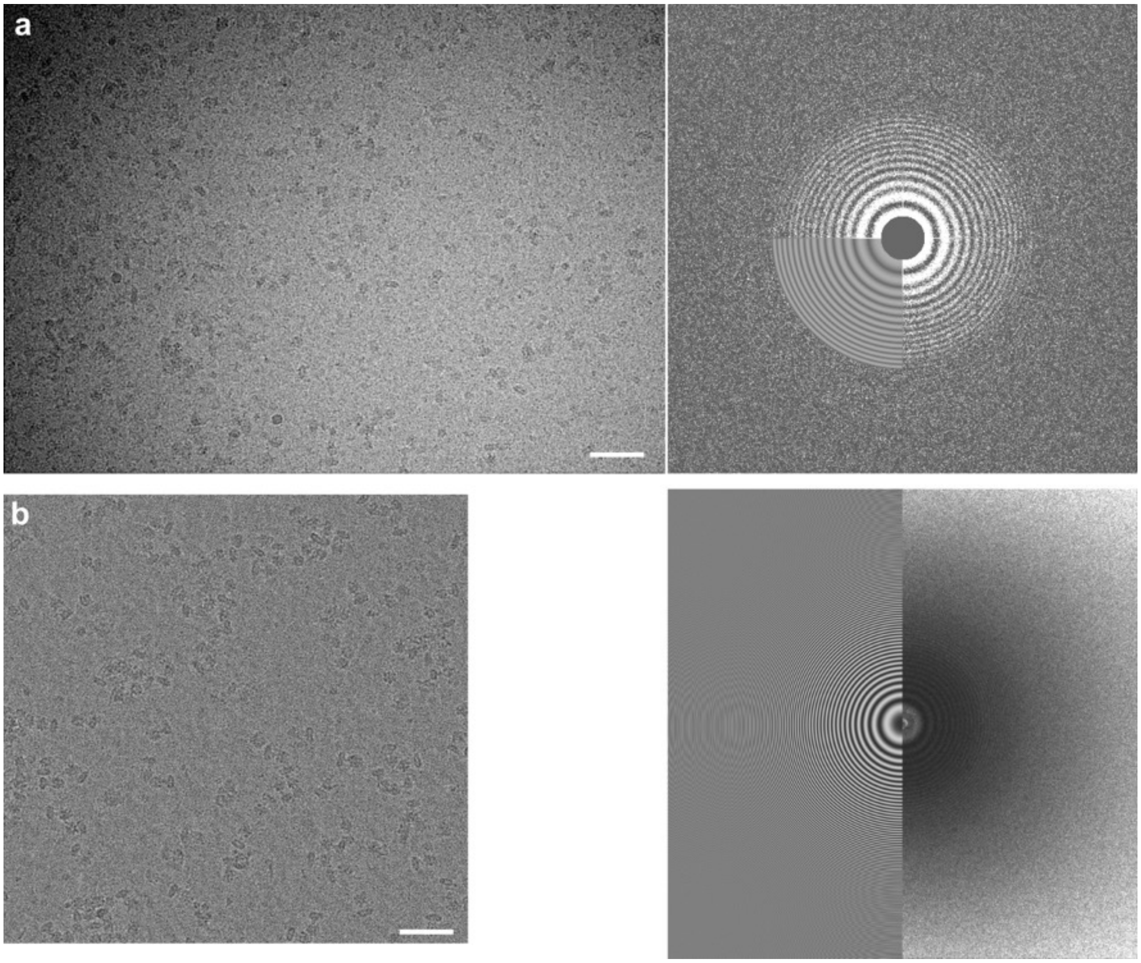

Supplementary Fig. 1: Cryo-EM micrographs and power spectrums. (**a**) WT *E. coli* F_1F_o ATP synthase. (**b**) εΔCTH *E. coli* F_1F_o ATP synthase. **(**left panels**)** Representative micrograph showing ATP synthase particles (white scale bar is equivalent to 50 nm) and (right panels) power spectrum of same micrograph. The data was taken on a distinct sample, we have imaged *E. coli* ATP synthase many times and taken full cryo-EM datasets >10 times. The micrographs shown were chosen to show clear ATP synthase particles (high defocus) and are devoid of ice contamination and particle aggregation.

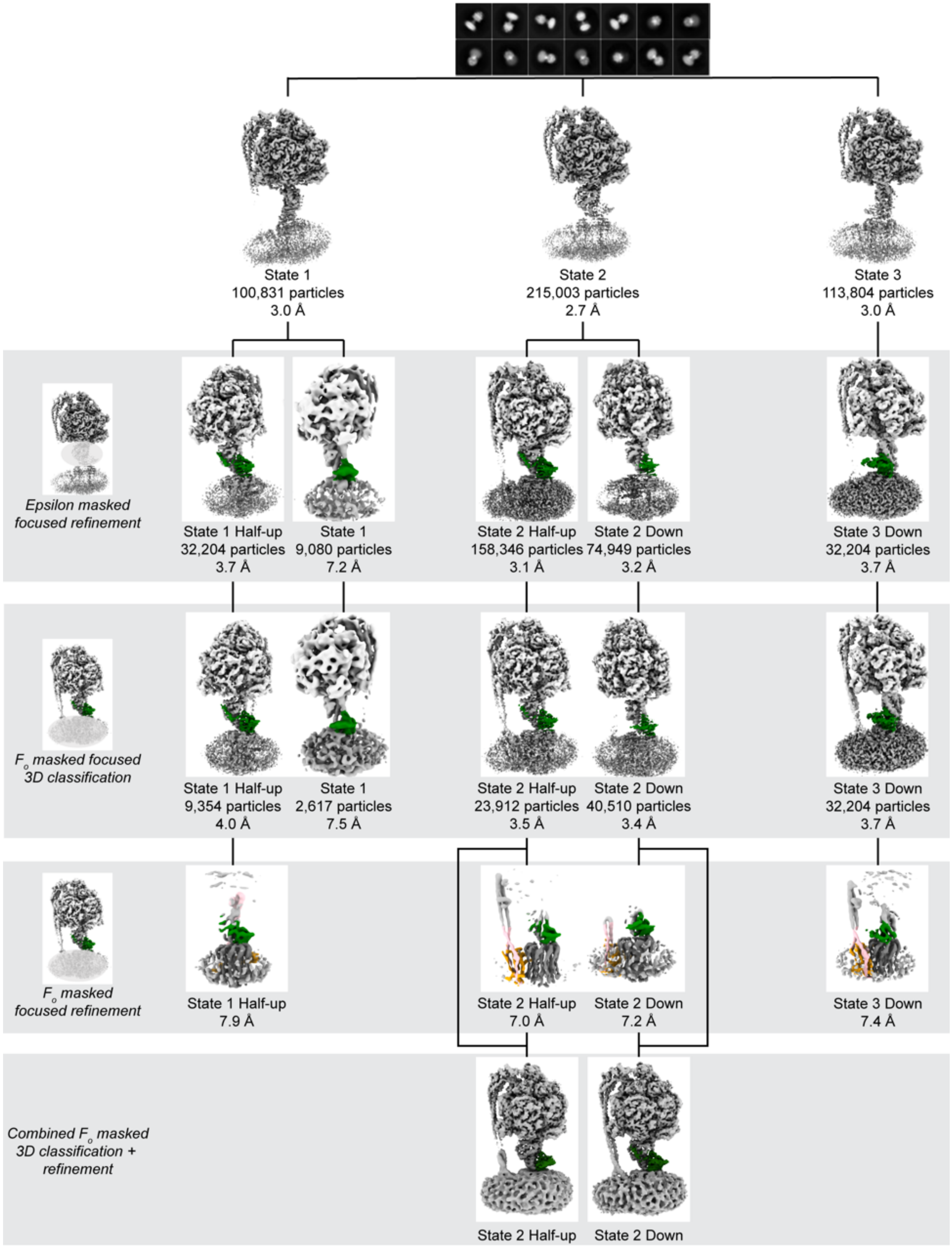

Supplementary Fig. 2: Flowchart describing the classification of particles into distinct conformations in the WT *E. coli* F_1F_o ATP synthase + 10 mM MgATP dataset.

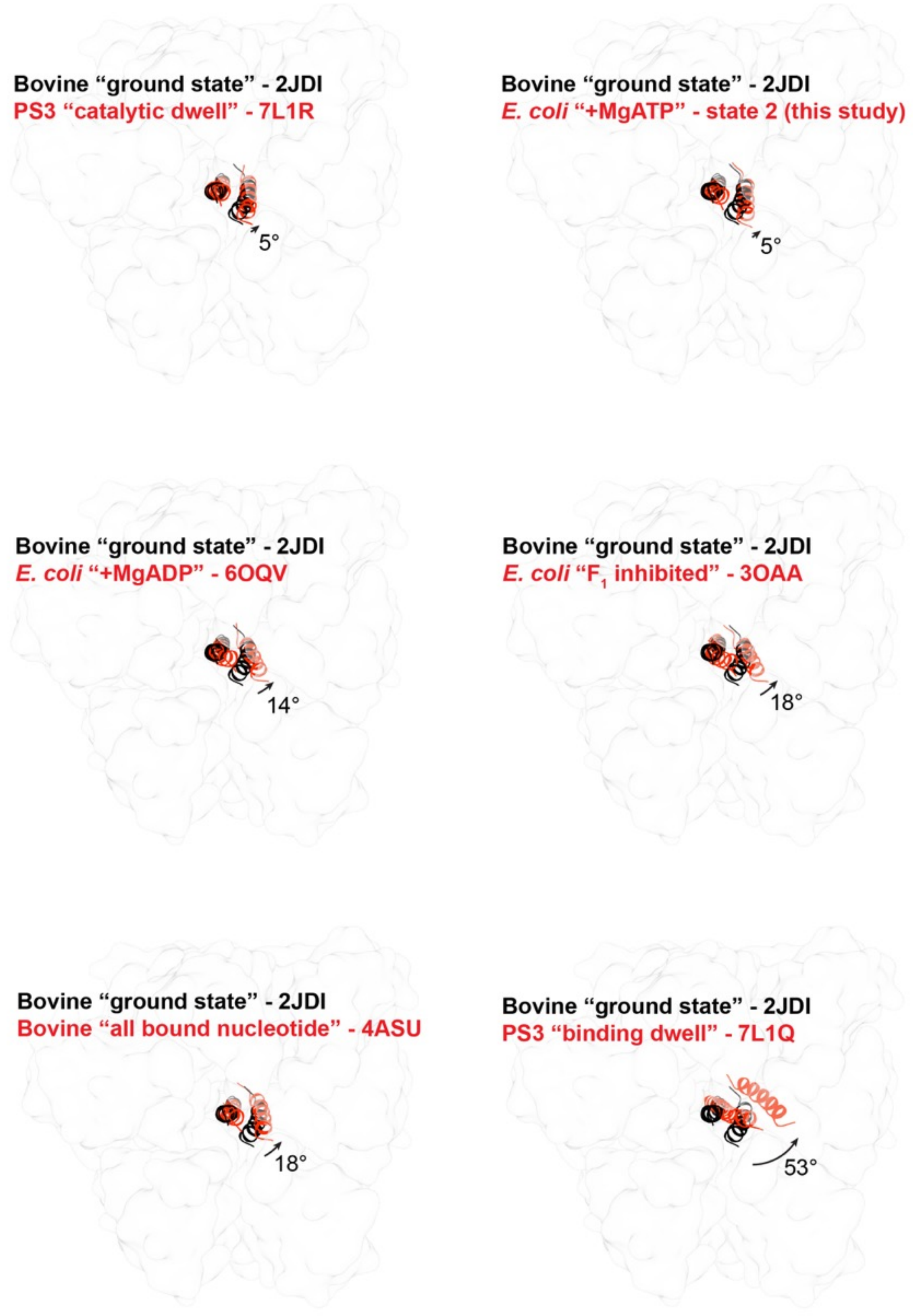

Supplementary Fig. 3: Comparison of the relative axel rotary position between F_1-ATPase structures. Structures were first aligned using the β barrel crown (residues α27-136 and β2-116) and then the rotation angle of the axel (bacterial residues 1-22 & 247-284; bovine residues 1-22 & 233-273) was calculated in Chimera. Bovine mitochondrial F_1-ATPase ground state in black and comparison structure in red.

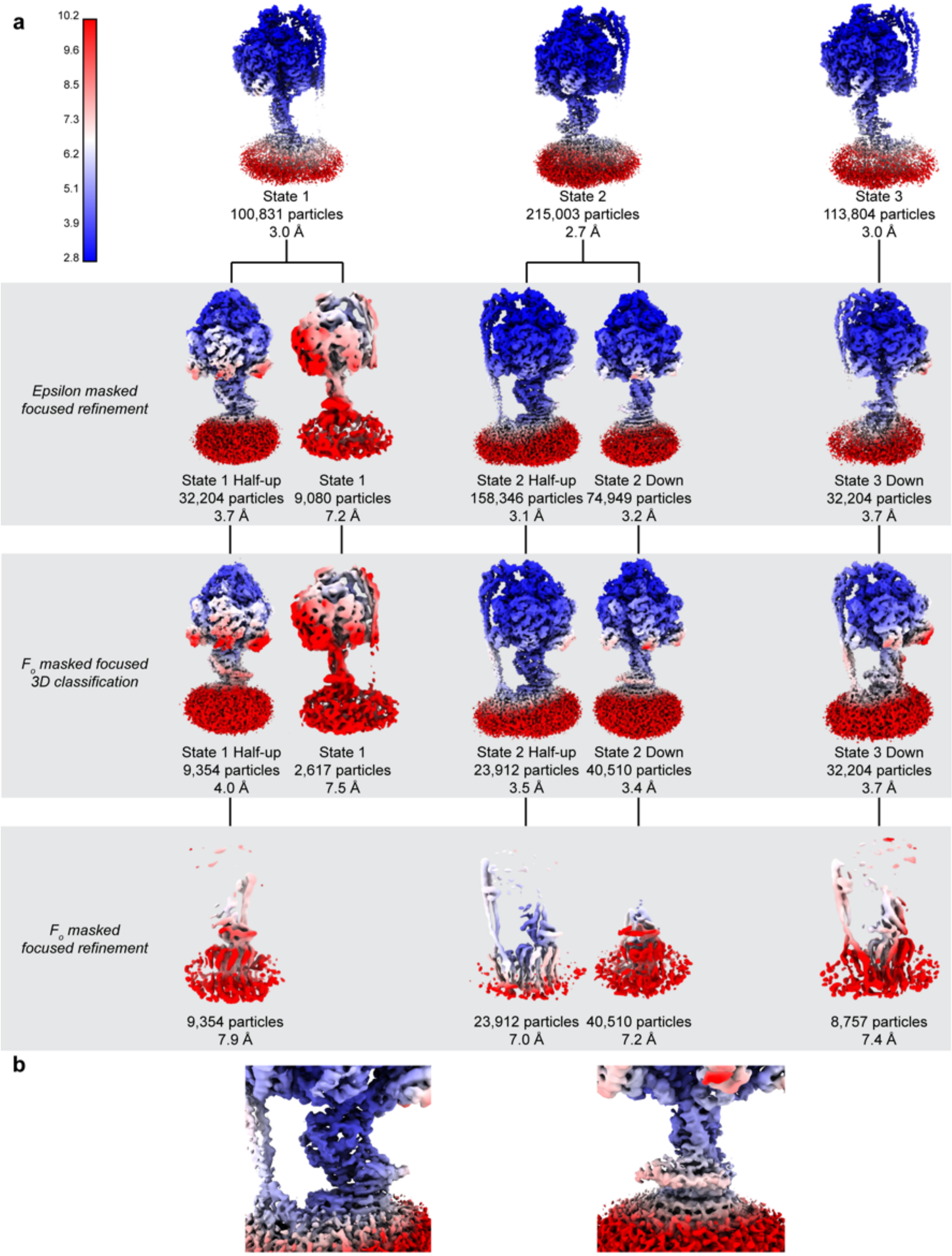

Supplementary Fig. 4: Local resolution estimates. Local resolution estimates of maps described in Supplementary Fig. 2 (**a**) and close ups of the central stalk region in State 2 "half-up" and "down" maps (**b**) to highlight resolution estimates in this region.

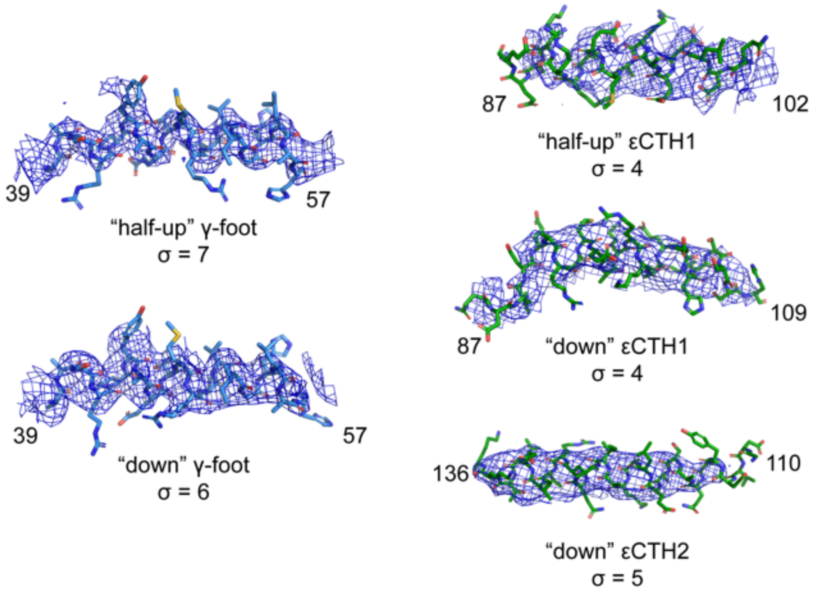

Supplementary Fig. 5: Cryo-EM maps of the γ foot and εCTD in the State 2 "half-up" and "down" sub states. Close up view of the cryo-EM maps corresponding to the γ foot (residues 39-57), εCTH1 (residues 87-102) and εCTH2 (residues 110-136).

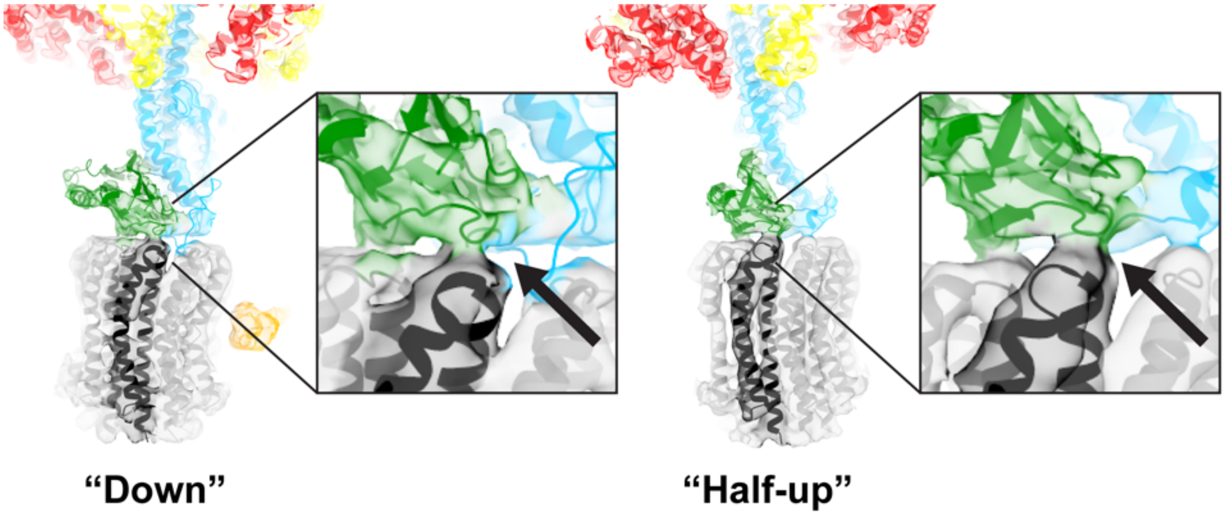

Supplementary Fig. 6: The interaction of the εNTD and c ring facilitates assignment of the c subunits. Model and maps of State 2 "down" (left) and "half-up" (right) show how relative c subunit rotation was assigned using the interaction with the εNTD (indicated with arrow).

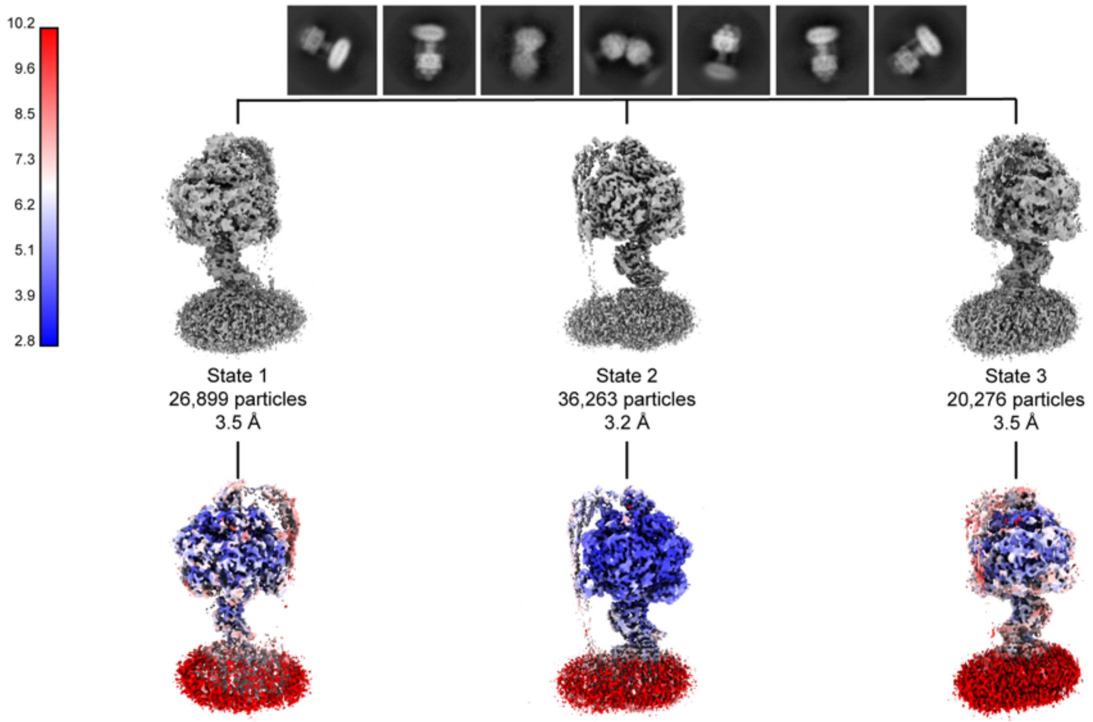

Supplementary Fig. 7: Flowchart describing the classification of particles into distinct conformations in the εΔCTH1 *E. coli* F_1F_o ATP synthase + 10 mM MgATP dataset.

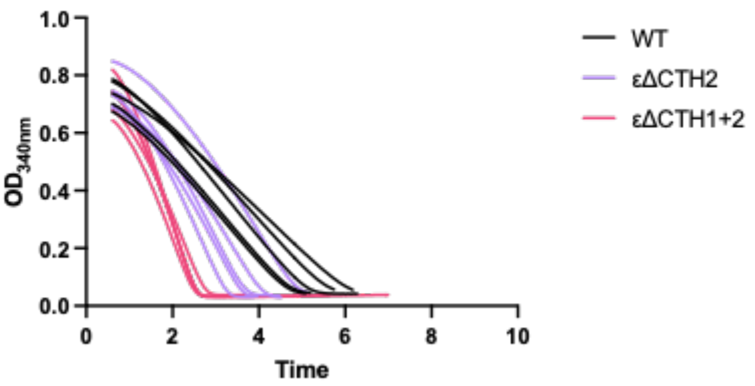

Supplementary Fig. 8: ATP regeneration assays. Raw trace for the ATP regeneration assays of WT, εΔCTH2 and εΔCTH1+2 mutations.

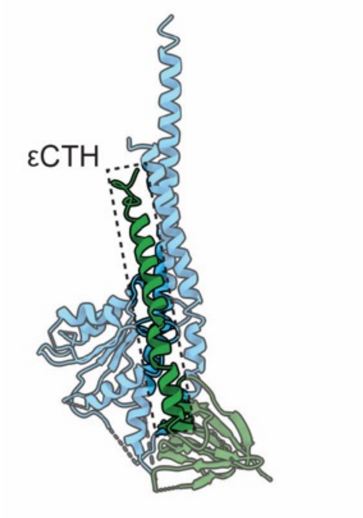

Supplementary Fig. 9: Subunits γ and ε from *Bacillus* PS3. Subunit ε shows a single extended εCTH (highlighted and labelled) that binds in a distinct manner compared to *E. coli* εCTD (shown in this study – see Fig. 1b). Model retrieved from PDB:4xd7[1].

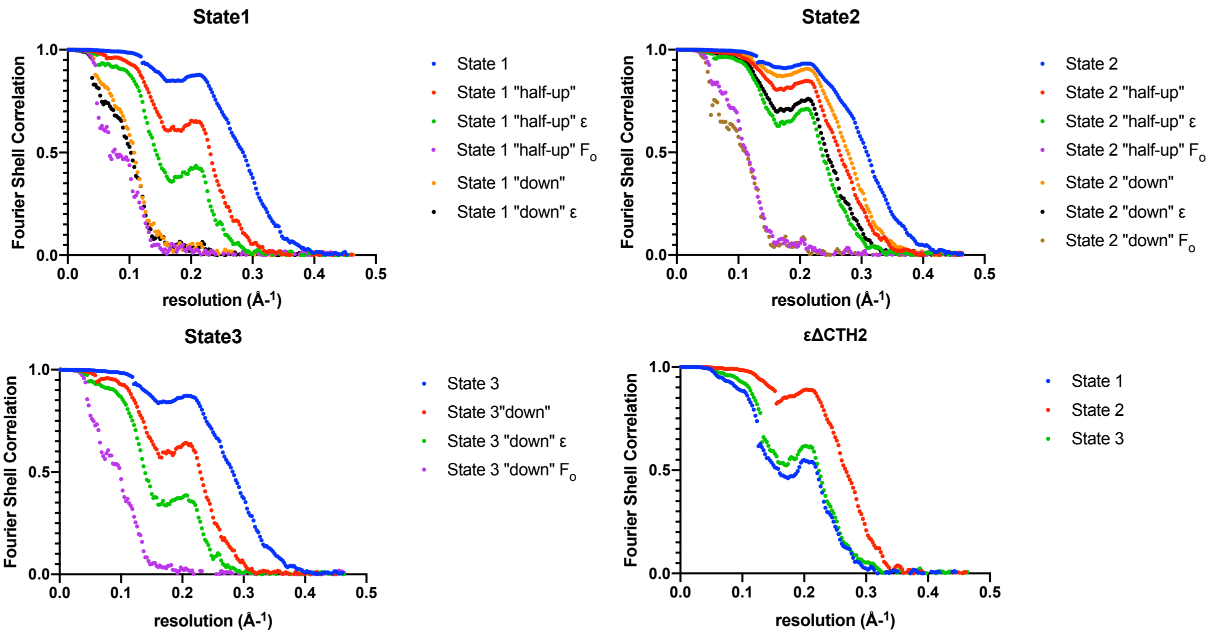

Supplementary Fig. 10: Fourier shell correlation curves for cryo-EM maps presented in this study.

	#1 State1 (EMD B 27296)	#2 State1 half-up (EMD B 27297) (PDB 8DBP)	#3 State1 half-up Fo classifi ed (EMD B 27298) (PDB 8DBQ)	#4 State1 half-up Fo refine (EMD B 27299)	#5 State1 down (EMD B 27300)	#6 State1 down Fo classifi ed (EMD B 27301)	#7 State2 558 (EMD B 27302)	#8 State2 half-up (EMD B 27303) (PDB 8DBR)	#9 State2 half-up Fo classifi ed (EMD B 27304) (PDB 8DBS)	#10 State2 half-up Fo refine (EMD B 27305)	#11 State2 down (EMD B 27306) (PDB 8DBT)	#12 State2 down Fo classifi ed (EMD B 27307) (PDB 8DBU)	#13 State2 down Fo refine (EMD B 27308)	#14 State3 (EMD B 27309)	#15 State 3 down (EMD B 27310) (PDB 8DBV)	#16 State 3 down Fo classifi ed (EMD B 27311) (PDB8 DBW)	#17 State 3 down Fo refine (EMD B 27312)	#18 State1 εΔCT H2 (EMD B 27313)	#19 State2 εΔCT H2 (EMD B 27314)	#20 State3 εΔCT H2 (EMD B 27315)
**Data collection and processing**																				
Magnification	81,000	81,000	81,000	81,000	81,000	81,000	81,000	81,000	81,000	81,000	81,000	81,000	81,000	81,000	81,000	81,000	81,000	81,000	81,000	81,000
Voltage (kV)	300	300	300	300	300	300	300	300	300	300	300	300	300	300	300	300	300	300	300	300
Electron exposure ($e^-/Å^2$)	48	48	48	48	48	48	48	48	48	48	48	48	48	48	48	48	48	48	48	48
Defocus range (μm)	0.8-3.5	0.8-3.5	0.8-3.5	0.8-3.5	0.8-3.5	0.8-3.5	0.8-3.5	0.8-3.5	0.8-3.5	0.8-3.5	0.8-3.5	0.8-3.5	0.8-3.5	0.8-3.5	0.8-3.5	0.8-3.5	0.8-3.5	0.8-3.5	0.8-3.5	0.8-3.5
Pixel size (Å)	1.08	1.08	1.08	1.08	1.08	1.08	1.08	1.08	1.08	1.08	1.08	1.08	1.08	1.08	1.08	1.08	1.08	1.08	1.08	1.08
Symmetry imposed	C1	C1	C1	C1	C1	C1	C1	C1	C1	C1	C1	C1	C1	C1	C1	C1	C1	C1	C1	C1
Initial particle images (no.)	429,63 8	429,63 8	429,63 8	429,63 8	429,63 8	429,63 8	429,63 8	429,63 8	429,63 8	429,63 8	429,63 8	429,63 8	429,63 8	429,63 8	429,63 8	429,63 8	429,63 8	83,440	83,440	83,440
Final particle images (no.)	100,83 1	33,587	9,354	9,254	9,080	2,617	215,00 3	74,946	23,917	23,917	159,24 6	40,510	40,510	113,80 4	32,204	8,757	8,757	26,899	36,263	20,276
Map resolution (Å) 0.143 FSC threshold	3.0	3.6	4.0	7.4	7.2	7.8	2.7	3.2	3.5	7.0	3.1	3.4	7.2	3.0	3.7	4.1	7.4	4.0	3.3	3.9
**Refinement**																				
Initial models used (PDB codes)	-	6OQT	6OQT	-	-	-	-	6OQV	6OQV	-	6OQV, 1AQT	6OQV, 1AQT	-	-	6OQW ,1AQT	6OQW ,1AQT	-	-	-	-
Model resolution (Å) 0.5 FSC threshold, masked/ unmasked	-	3.7/4.2	4.2/6.6	-	-	-	-	3.2/3.6	3.6/4.0	-	3.2/3.4	3.5/3.9	-	-	3.8/4.2	4.2/6.7	-	-	-	-
Map sharpening *B* factor ($Å^2$)	-57	-62	-73	-169	-125	-114	-53	-61	-63	-185	-63	-53	-199	-58	-74	-75	-176	-14.8	-38.6	-22.4
Model composition																				
Non-hydrogen atoms	-	36,632	36,737	-	-	-	-	36,678	36,678	-	36,937	36.917	-	-	36,863	36,839	-	-	-	-
Protein residues	-	4,800	4,819	-	-	-	-	4,814	4,814	-	4,847	4,845	-	-	4,837	4,837	-	-	-	-
Ligands	-	11	11	-	-	-	-	11	11	-	11	11	-	-	11	11	-	-	-	-
B factors ($Å^2$)																				
Protein	-	148.21	173.69	-	-	-	-	159.65	131.51	-	219.08	137.28	-	-	145.65	207.29	-	-	-	-
Ligand	-	87.60	111.04	-	-	-	-	85.50	66.60	-	86.12	64.63	-	-	64.93	116.50	-	-	-	-
R.m.s. deviations																				
Bond lengths (Å)	-	0.01	0.005	-	-	-	-	0.010	0.004	-	0.01	0.004	-	-	0.010	0.004	-	-	-	-
Bond angles (°)	-	1.207	0.892	-	-	-	-	1.287	0.640	-	1.267	0.837	-	-	1.231	0.811	-	-	-	-
Validation																				
MolProbity score	-	1.23	1.04	-	-	-	-	1.20	1.48	-	1.18	1.05	-	-	1.52	1.16	-	-	-	-
Clashscore	-	2.42	1.98	-	-	-	-	2.71	4.94	-	2.49	2.60	-	-	3.33	2.77	-	-	-	-
Poor rotamers (%)	-	1.39	1.26	-	-	-	-	0.74	1.94	-	0.65	0.76	-	-	1.86	1.10	-	-	-	-
Ramachandran plot																				
Favored (%)	-	97.52	98.35	-	-	-	-	97.25	98.43	-	97.19	98.40	-	-	97.08	97.70	-	-	-	-
Allowed (%)	-	2.38	1.63	-	-	-	-	2.68	1.53	-	2.77	1.58	-	-	2.88	2.23	-	-	-	-
Disallowed (%)	-	0.11	0.02	-	-	-	-	0.06	0.04	-	0.04	0.02	-	-	0.04	0.06	-	-	-	-

Supplementary Table 1: Cryo-EM data collection, refinement, and validation statistics.

References

1. Shirakihara, Y. *et al.* Structure of a thermophilic F_1-ATPase inhibited by an epsilon-subunit: deeper insight into the epsilon-inhibition mechanism. *FEBS J* **282**, 2895-2913, doi:10.1111/febs.13329 (2015).
