# Supplementary material for: Changes within the central stalk of E. coli F1Fo ATP synthase observed after addition of ATP
Source: Commun Biol. 2023 Jan 11;6:26. doi: 10.1038/s42003-023-04414-z (PMC9834311; doi:10.1038/s42003-023-04414-z)
Supplement: Supplementary file 3 — Description of Additional Supplementary Files [file 42003_2023_4414_MOESM3_ESM.pdf]

Description of Additional Supplementary Files

File name: Supplementary Data 1
Description: PDB atomic model - State 2 down (composite map).

File name: Supplementary Data 2
Description: MRC map - State 2 down.

File name: Supplementary Data 3
Description: PDB atomic model - State 2 half up (composite map).

File name: Supplementary Data 4
Description: MRC map - State 2 half up.

File name: Supplementary Data 5
Description: Uncropped SDS PAGE in Figure 7d.

File name: Supplementary Data 6
Description: Values of chart in Figure 7e.
