# Supplementary figures and images for: Changes within the central stalk of E. coli F1Fo ATP synthase observed after addition of ATP
Source: Commun Biol. 2023 Jan 11;6:26. doi: 10.1038/s42003-023-04414-z (PMC9834311; doi:10.1038/s42003-023-04414-z)

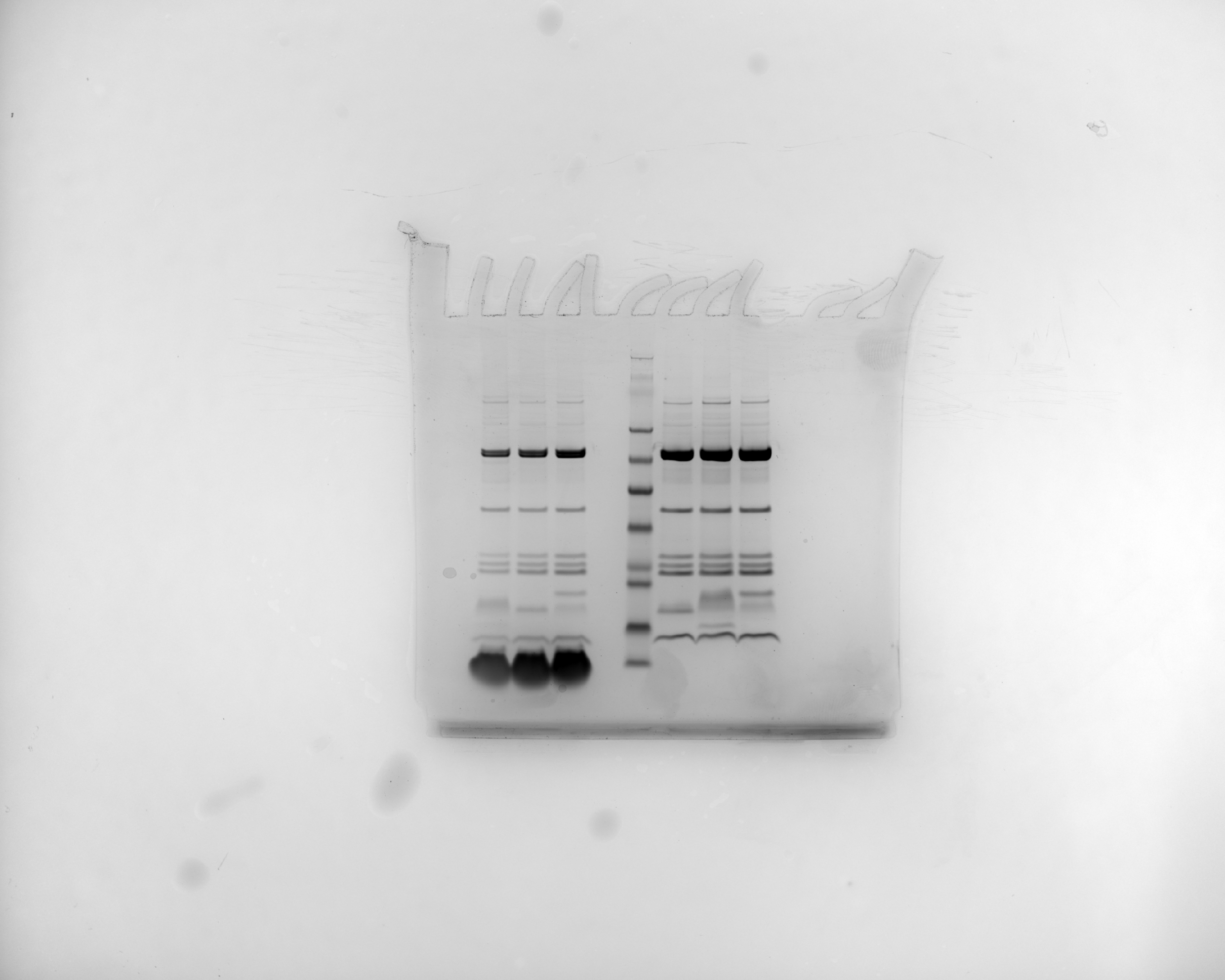

Supplement: Supplementary file 8 — Supplementary Data 5 [file 42003_2023_4414_MOESM8_ESM.tif]
