# Supplementary material for: Changes within the central stalk of E. coli F1Fo ATP synthase observed after addition of ATP
Source: Commun Biol. 2023 Jan 11;6:26. doi: 10.1038/s42003-023-04414-z (PMC9834311; doi:10.1038/s42003-023-04414-z)
Supplement: Supplementary file 10 — Reporting Summary [file 42003_2023_4414_MOESM10_ESM.pdf]

nature portfolio

Corresponding author(s): Alastair Stewart
Last updated by author(s): Dec 10, 2022

Reporting Summary

Nature Portfolio wishes to improve the reproducibility of the work that we publish. This form provides structure for consistency and transparency in reporting. For further information on Nature Portfolio policies, see our Editorial Policies and the Editorial Policy Checklist.

Statistics

For all statistical analyses, confirm that the following items are present in the figure legend, table legend, main text, or Methods section.

n/a	Confirmed	
☐	☒	The exact sample size (*n*) for each experimental group/condition, given as a discrete number and unit of measurement
☐	☒	A statement on whether measurements were taken from distinct samples or whether the same sample was measured repeatedly
☒	☐	The statistical test(s) used AND whether they are one- or two-sided<br>*Only common tests should be described solely by name; describe more complex techniques in the Methods section.*
☒	☐	A description of all covariates tested
☒	☐	A description of any assumptions or corrections, such as tests of normality and adjustment for multiple comparisons
☐	☒	A full description of the statistical parameters including central tendency (e.g. means) or other basic estimates (e.g. regression coefficient) AND variation (e.g. standard deviation) or associated estimates of uncertainty (e.g. confidence intervals)
☒	☐	For null hypothesis testing, the test statistic (e.g. *F*, *t*, *r*) with confidence intervals, effect sizes, degrees of freedom and *P* value noted<br>*Give P values as exact values whenever suitable.*
☒	☐	For Bayesian analysis, information on the choice of priors and Markov chain Monte Carlo settings
☒	☐	For hierarchical and complex designs, identification of the appropriate level for tests and full reporting of outcomes
☒	☐	Estimates of effect sizes (e.g. Cohen's *d*, Pearson's *r*), indicating how they were calculated

Our web collection on statistics for biologists contains articles on many of the points above.

Software and code

Policy information about availability of computer code

Data collection	SerialEM: v3.7 and EPU:v2.7
Data analysis	MotionCorr2:v1.2.1, Gctf:v1.06, RELION:v3.0.6, cryoSPARC:v2.15.0, Coot:v0.9, PHENIX:v1.15-3459, ISOLDE:v1.0b3, cryoSPARC:v2.15.0, Prism 8 for macOS

For manuscripts utilizing custom algorithms or software that are central to the research but not yet described in published literature, software must be made available to editors and reviewers. We strongly encourage code deposition in a community repository (e.g. GitHub). See the Nature Portfolio guidelines for submitting code & software for further information.

Data

Policy information about availability of data

All manuscripts must include a data availability statement. This statement should provide the following information, where applicable:

- Accession codes, unique identifiers, or web links for publicly available datasets
- A description of any restrictions on data availability
- For clinical datasets or third party data, please ensure that the statement adheres to our policy

The models generated and analyzed during the current study are available from the RCSB PDB: 8DBP, 8DBQ, 8DBR, 8DBS, 8DBT, 8DBU, 8DBV, 8DBW. The cryo-EM maps used to generate models are available from the EMDB: 27296, 27297, 27298, 27299, 27300, 27301, 27302, 27303, 27304, 27305, 27306, 27307, 27308,

27309, 27310, 27311, 27312, 27313, 27314, 27315. Phenix combined maps and models for State 2 up and down are provided as Supplementary Data 1-4 with this manuscript.

Human research participants

Policy information about studies involving human research participants and Sex and Gender in Research.

Reporting on sex and gender	N/A
Population characteristics	N/A
Recruitment	N/A
Ethics oversight	N/A

Note that full information on the approval of the study protocol must also be provided in the manuscript.

Field-specific reporting

Please select the one below that is the best fit for your research. If you are not sure, read the appropriate sections before making your selection.

☒ Life sciences ☐ Behavioural & social sciences ☐ Ecological, evolutionary & environmental sciences

For a reference copy of the document with all sections, see nature.com/documents/nr-reporting-summary-flat.pdf

Life sciences study design

All studies must disclose on these points even when the disclosure is negative.

Sample size	The sample sizes (8,620/3,680 micrographs and 429,638/83,438 particles) was chosen to identify the differences between states reliably. This number is general practice in our field (e.g. Murphy, B. J. et al. Rotary substates of mitochondrial ATP synthase reveal the basis of flexible F1-Fo coupling, Science 364 (2019)).
Data exclusions	Images were excluded based on their CTF parameters (resolution fit to 5 Å or better), this was to ensure only high-quality images were used and that motion correction had been successful. Particles were excluded based on their general appearance and the number of particles in each set. Exclusion criteria were pre-established, in that we would not select “bad” particles or states that refined poorly. This is general practice in our field (e.g. Scheres S.H.W. “RELION: Implementation of a Bayesian approach to cryo-EM structure determination.” Journal of Structural Biology 2012 and Scheres S.H.W. “Chapter Six - Processing of Structurally Heterogeneous Cryo-EM Data in RELION.” Methods in Enzymology 2016).
Replication	As single particle analysis is an averaging method it is not usual to perform replicates, and hence no replication was performed (though the sample was screened many times at 200 kV showing similar results). This is due, in part, to extraordinary demand on 300 kV microscope time and the cost associated with experiments.
Randomization	Relion uses a reference free algorithm for 2D classification that starts with all particles in random sets. Randomization, other than this, is not used in this type of study (Scheres S.H.W. “RELION: Implementation of a Bayesian approach to cryo-EM structure determination.” Journal of Structural Biology 2012).
Blinding	This study was performed on a single protein sample. Blinding of the data would not have been feasible. Furthermore, computer classification was used to identify the sub-states, which addresses the possibility of operator bias.

Reporting for specific materials, systems and methods

We require information from authors about some types of materials, experimental systems and methods used in many studies. Here, indicate whether each material, system or method listed is relevant to your study. If you are not sure if a list item applies to your research, read the appropriate section before selecting a response.

Materials & experimental systems

n/a	Involved in the study
☒	☐ Antibodies
☒	☐ Eukaryotic cell lines
☒	☐ Palaeontology and archaeology
☒	☐ Animals and other organisms
☒	☐ Clinical data
☒	☐ Dual use research of concern

Methods

n/a	Involved in the study
☒	☐ ChIP-seq
☒	☐ Flow cytometry
☒	☐ MRI-based neuroimaging
